# Identification of the UDP-glucose-4-epimerase required for galactofuranose biosynthesis and galactose metabolism in *A. niger*

**DOI:** 10.1186/s40694-014-0006-7

**Published:** 2014-10-14

**Authors:** Joohae Park, Boris Tefsen, Mark Arentshorst, Ellen Lagendijk, Cees AMJJ van den Hondel, Irma van Die, Arthur FJ Ram

**Affiliations:** 1 Institute of Biology Leiden, Molecular Microbiology and Biotechnology, Leiden University, Sylviusweg 72, Leiden, 2333 BE The Netherlands; 2 Department of Molecular Cell Biology and Immunology, VU University Medical Center, van den Boechorststraat 7, Amsterdam, 1081 BT The Netherlands; 3 Present Address: Department of Biological Sciences, Xi’an Jiaotong Liverpool University, 111 Ren Ai Road, Dushu Lake Higher Education Town, Suzhou Industrial Park, Suzhou, 215123 Jiangsu, China

**Keywords:** Filamentous fungi, Cell wall, Cell wall integrity, Galactofuranose, Illumina sequencing, Whole genome sequencing, SNP analysis, UDP-glucose-4-epimerase, Mutant screen, Galactose

## Abstract

**Background:**

Galactofuranose (Gal*f*)-containing glycoconjugates are important to secure the integrity of the cell wall of filamentous fungi. Mutations that prevent the biosynthesis of Gal*f*-containing molecules compromise cell wall integrity. In response to cell wall weakening, the cell wall integrity (CWI)-pathway is activated to reinforce the strength of the cell wall. Activation of CWI-pathway in *Aspergillus niger* is characterized by the specific induction of the *agsA* gene, which encodes a cell wall α-glucan synthase.

**Results:**

In this study, we screened a collection of cell wall mutants with an induced expression of *agsA* for defects in Gal*f* biosynthesis using a with anti-Gal*f* antibody (L10). From this collection of mutants, we previously identified mutants in the UDP-galactopyranose mutase encoding gene (*ugmA*). Here, we have identified six additional UDP-galactopyranose mutase (*ugmA*) mutants and one mutant (named mutant #41) in an additional complementation group that displayed strongly reduced Gal*f*-levels in the cell wall. By using a whole genome sequencing approach, 21 SNPs in coding regions were identified between mutant #41 and its parental strain which changed the amino acid sequence of the encoded proteins. One of these mutations was in gene An14g03820, which codes for a putative UDP-glucose-4-epimerase (UgeA). The A to G mutation in this gene causes an amino acid change of Asn to Asp at position 191 in the UgeA protein. Targeted deletion of *ugeA* resulted in an even more severe reduction of Gal*f* in N-linked glucans, indicating that the UgeA protein in mutant #41 is partially active. The *ugeA* gene is also required for growth on galactose despite the presence of two UgeA homologs in the *A. niger* genome.

**Conclusion:**

By using a classical mutant screen and whole genome sequencing of a new Gal*f*-deficient mutant, the UDP-glucose-4-epimerase gene (*ugeA*) has been identified. UgeA is required for the biosynthesis of Gal*f* as well as for galactose metabolism in *Aspergillus niger*.

**Electronic supplementary material:**

The online version of this article (doi:10.1186/s40694-014-0006-7) contains supplementary material, which is available to authorized users.

## Background

The cell wall is an essential component of the fungal cell. Cells can survive the enzymatic removal of the cell wall but the resulting protoplasts need to be stabilized in an environment with high osmolarity to withstand internal turgor pressure. The cell wall of filamentous fungi consists mainly (90%) of polysaccharide material, including polymers of glucose (β-1,3- and β-1,6-glucans, α-1,3-glucan), *N*-acetylglucosamine (chitin), mannose and galactofuranose (galactomannan), galactoaminogalactan and of about 10% of cell wall glycoproteins (galactomanno-proteins) [1]–[3]. The different glycoconjugates are either synthesized at the plasma membrane by specific cell membrane-localized synthases (e.g. chitin, β-1,3- and α-1,3-glucan) or preassembled in the secretory pathway (galactomannan and galactomannoproteins). During or after transport over the membrane, polymers are cross-linked with each other via covalent or hydrogen bonds to create a sturdy cell wall.

We and others have previously shown that galactofuranose (Gal*f*) is an important component of the cell wall in *Aspergillus* species. It is found in several glycoconjugates including galactomannan, secreted and cell wall proteins via *N*- and *O*-linked chains, and glycosphingolipids (see for review [4]). A key enzyme in Gal*f* biosynthesis is UDP-Gal*p* mutase (UgmA), which converts the pyranose form of UDP-galactose (UDP-Gal*p*) into UDP-Gal*f*. Only UDP-Gal*f* can be transported into the Golgi where Gal*f* is used as a donor sugar for the synthesis of Gal*f*-containing structures [5]–[7]. Gene disruption approaches of the *ugmA* gene in *Aspergillus niger, A. fumigatus, A. nidulans* have shown that Gal*f* biosynthesis is required for hyphal morphogenesis and cell wall architecture [8]–[10], whereas disruption of the *ugmA* homolog in *Cryptococcus neoformans* did not have an apparent growth phenotype nor did it affect capsule formation [11]. Inactivation of the *ugmA* genes in Aspergilli results in an increased sensitivity towards cell wall assembly interfering drugs such as calcofluor white (CFW), indicating that galactofuranose-containing glycoconjugates are necessary for maintaining the integrity of the cell wall [8].

In *A. niger* the *ugmA* gene was identified in a screen for mutants in which the cell wall stress reporter gene *agsA* was constitutively induced [8]. Besides UgmA, proteins required for the biosynthesis of Gal*f*-containing glycoconjugates have been identified in both *A. nidulans* and *A. fumigatus*. These proteins include UgeA/Uge5, encoding the UDP-glucose-4-epimerase necessary for the synthesis of UDP-Gal*p* [12],[13], UgtA/GlfB encoding a Golgi localized UDP-Gal*f* transporter protein [5],[6] and GfsA encoding a Gal*f* transferase [7].

In this study, we have screened a collection of 240 cell wall mutants with induced *agsA* expression [8] for mutants that do not secrete Gal*f* containing glycoconjugates into the growth medium. In addition to a large complementation group of 9 *ugmA* mutants, one additional Gal*f*-low mutant (#41), belonging to a different complementation group, was identified. Whole genome sequencing of this mutant revealed that the newly identified Gal*f*-mutant contains a mutation in gene An14g03820. This gene is predicted to encode a putative UDP-glucose-4-epimerase gene (*ugeA*), required for the biosynthesis of Gal*f* as well as for Gal*p* metabolism in *Aspergillus niger*.

## Results

### Screening of Gal*f*-deficient mutants within 240 *A. niger* cell wall mutants

**Figure 1:**
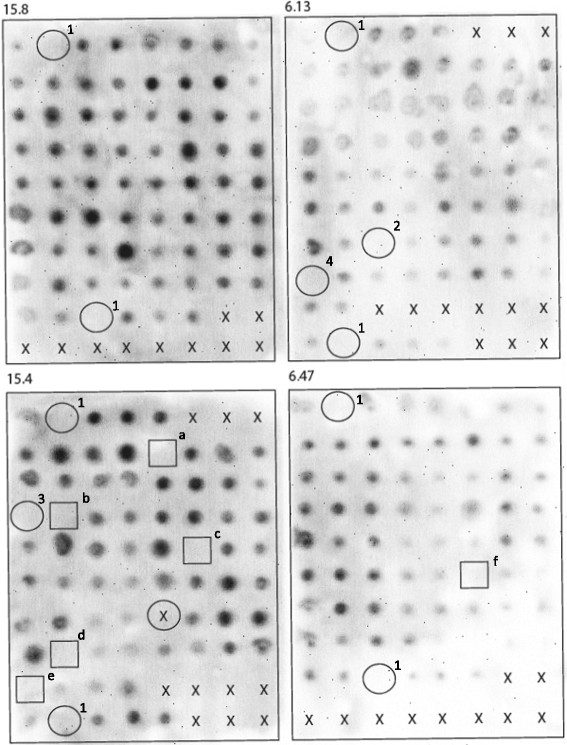
**Dot blot assay to detect the presence of Gal** ***f*** **residues on secreted glycoconjugates from** ***A. niger*** **mutants.** 240 *A. niger* cell wall mutant strains were grown to early stationary phase and cell-free medium was spotted on nitrocellulose filter paper. The blots were incubated with the anti-Gal*f* antibody (L10) to detect the presence of Gal*f.* Places where no medium was spotted are indicated with X; open circles indicate known Gal*f*-minus mutants (1 = *ΔugmA*, 2 = 6.13#44/miaA, 3 = 15.4#17/miaB, 4 = 6.13#50/miaC); a mutant that did not grow in this experiment is indicated with X in an open circle; open squares indicate newly detected Gal*f*-minus mutants (a = 15.4#5, b = 15.4#18, c = 15.4#30, d = 15.4#50, e = 15.4#57 and f = 6.47#41). 15.8, 6.13, 15.4 and 6.47 on top of each blot indicate the different parental backgrounds in which the mutants were made.

**Figure 2:**
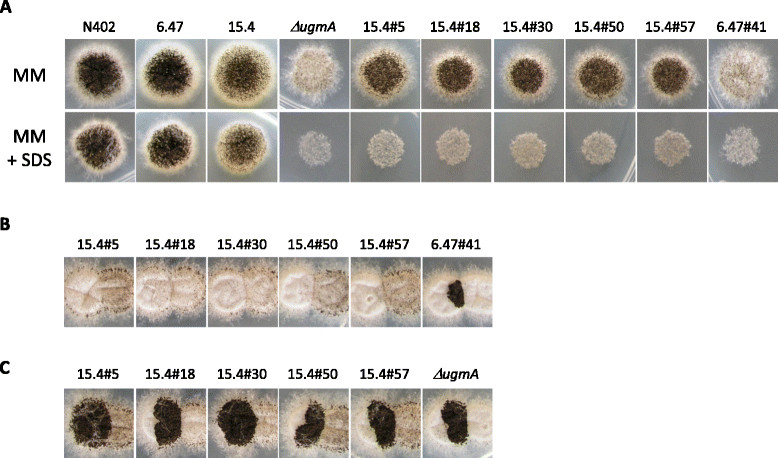
**Mutant 6.47#41 belongs to a different complementation group as** ***ΔugmA*** **mutants. A)** Gal*f*-deficient mutants display a reduced conidiation phenotype. The reduced conidiation is more pronounced on plates containing 0.005% SDS. **B)** Heterokaryon formation of newly identified Gal*f*-minus mutants (left colony) with the *ΔugmA* mutant (right colony). **C)** Heterokaryon formation of newly identified Gal*f*-minus mutants (left colony) with mutant 6.47#41 (right colony). Strains were grown for 5 days at 30° on MM containing 0.005% SDS.

To identify additional genes involved in the biosynthesis of cell wall galactomannan in *A. niger*, we screened a collection of 240 cell wall mutants for Gal*f*-deficient strains. Gal*f* containing structures such as galactomannans and *N*- or *O*-glycosylated proteins are secreted in the medium and therefore analysis of the medium for the presence of Gal*f* using an antibody can identify Gal*f* minus mutants. From the collection of cell wall mutants, we previously identified the *A. niger* UDP-galactopyranose mutase (UgmA) as an essential protein for the formation of Gal*f*-containing cell wall glycoconjugates ([8], see below). The selection of the three *ugmA* mutants in that study was not based on their Gal*f* phenotype, but on their Calcofluor white- and SDS-hypersensitive phenotype. Here, all 240 mutants were grown in liquid medium and 2 μl of medium was used in a dot blot analysis using the anti-Gal*f* antibody L10 [14] as described in Materials and Methods (Figure 1). The screening confirmed the absence of Gal*f* in the three *ugmA* mutants already identified (6.13#44, 15.4#17, and 6.13#50) [8], and identified six additional Gal*f*-minus mutants (15.4#30, 15.4#18, 15.4#5, 15.4#50, 15.4#57 and 6.47#41). A heterokaryon complementation test using the *ΔugmA* strain was performed to determine whether the newly identified mutants were also mutated in *ugmA*. In this test, spores of mutants of interest are inoculated close to each other. Outgrowth of the spores will result in the formation of heterokaryotic mycelium in the contact zones. The phenotype of all Gal*f*-deficient mutants identified was characterized by a reduced sporulation (Figure 2A) and this phenotype was used to assay complementation. As shown in Figure 2B only mutant #41 was complemented by the *ΔugmA* mutant, which is visible by a well sporulating zone of heterokaryotic mycelium in the contact zones of the two colonies. The other mutants were not complemented by the *ΔugmA* mutant (data not shown) and therefore considered to be mutant alleles of *ugmA*. In contrast, mutant #41 showed a clear zone of sporulation in the contact zones with the previously identified *ΔugmA* mutant (Figure 2B) as well as with the *ΔugmA* mutants identified in this study (Figure 2C), indicating that the *ΔugmA* mutant and #41 mutant are in two different complementation groups. To confirm that the #41 mutant was not mutated in the *ugmA* gene, the *ugmA* locus of the #41 mutant was amplified by PCR and sequenced. No mutations in the *ugmA* gene were found.

### Mutant #41 has reduced levels of Gal*f*

**Figure 3:**
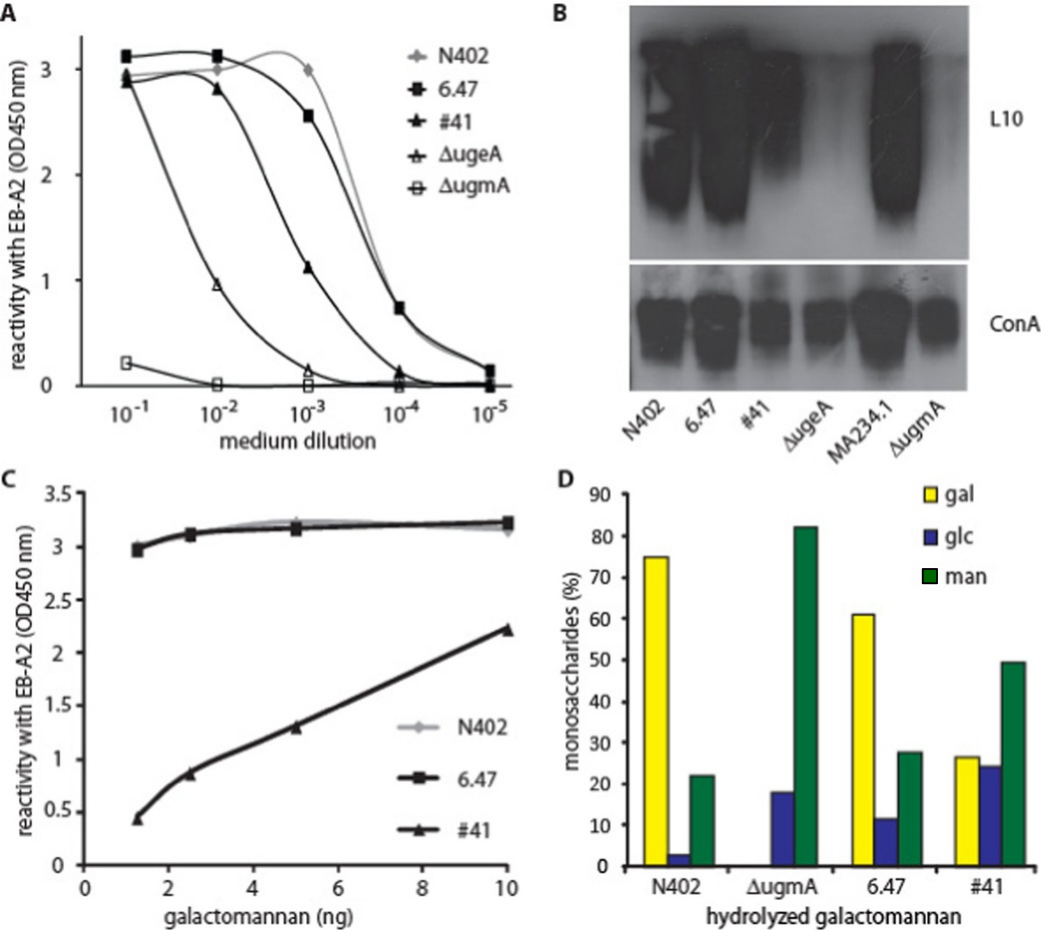
**Gal** ***f*** **in mutant #41 is greatly reduced but not absent. A)** Platelia assay with anti-Gal*f* antibody EB-A2 to detect Gal*f* residues in a dilution range of medium samples from the indicated strains **B)** Western blot analysis with anti-Gal*f* antibody L10 on medium samples from the indicated strains (upper blot) and ConA lectin (lower blot) **C)** Platelia assay on purified galactomannan to analyse the presence of Gal*f* **D)** Galactomannan from the indicated strains was hydrolyzed and their sugar composition was analyzed by HPAEC and depicted here as a percentage of the total amount of sugar in each sample.

To address the Gal*f-* deficient phenotype of the #41 mutant further, medium samples of mutant #41 were analysed using the Platelia assay. This quantitative assay for Gal*f* detection uses the monoclonal EB-A2 antibody which recognizes Gal*f*-moieties on galactomannoproteins [15]. As shown in Figure 3A, titration of the medium samples revealed an approximately 10-fold reduced amount of Gal*f*-reactive epitopes produced by the #41 mutant compared to that of its parental strain RD6.47. As controls, high reactivity with medium from wild type N402 and no reactivity with medium from the *ΔugmA* strain were measured in this assay. Western blot analysis of extracellular medium proteins using the L10 antibody also detected less Gal*f* epitopes on secreted glycoproteins in contrast to the wild type strains N402 and RD6.47, but clearly more than in the medium of the *ΔugmA* strain (Figure 3B). Detection of *N*-glycans with the lectin ConcavalinA (ConA) was performed as a control.

To obtain more insight into the presence of Gal*f* in the cell wall of the #41 mutant, galactomannan from two Gal*f* mutants (*ΔugmA* and #41), and the wild-type strain (N402) was isolated essentially as described previously by Bardalaye and Nordin, 1977 [16] (see Methods for details). A titration of the purified galactomannan fraction was applied to the Platelia assay, revealing again a lowered reactivity of the Gal*f*-antibody with the galactomannan derived from #41 compared to the wild-type strain (Figure 3C), whereas the polysaccharide isolated from *ΔugmA* was not reactive at all. This indicated that there are less Gal*f*-moieties present on the galactomannan from #41 compared to wild type galactomannan. To confirm this result, the monosaccharide composition of the galactomannan fraction of these strains was analysed by HPAEC analysis (DIONEX system) after hydrolysis (Figure 3D). As expected, no Gal*f* was detected in the hydrolyzed polysaccharide isolated from the *ΔugmA* mutant. The ratio Gal*f* /Man of the wild-type strains was found to be well above 2 (2.2-2.8), while this ratio was only 0.5 for the #41 mutant. These results demonstrate that the amount of Gal*f* is reduced more than 4-fold in the #41 mutant compared to its parent strain. This lowered amount of Gal*f* apparently has escaped detection by the L10 antibody in our initial screening by dot blot, leading to its discovery (Figure 1), but clearly is detected by the Platelia assay (Figure 3A and 3C), using the EB-A2 antibody, as well as by Western blot analysis using the L10 antibody (Figure 3B).

### Genomic characterization of mutant #41 by whole genome sequencing

An *A. niger* genomic cosmid library [17] was used in several unsuccessful attempts to complement the reduced sporulating growth phenotype of mutant #41. As an alternative approach, the genome of both the parental strain (RD6.47) and the #41 mutant were sequenced by pair-end Illumina sequencing. A single nucleotide polymorphism (SNP) analysis between the two strains was performed and 78 SNPs were identified (Additional file 1: Table S1). These SNPs were analysed further by determining whether a particular SNP was located in a predicted ORF and whether this SNP affected the amino acid sequence. We identified 21 SNPs in genes in the #41 mutant that caused changes in the amino acid sequence (Additional file 2: Table S2). Most of these SNPs seemed unrelated to Gal*f*-biosynthesis, but the mutation in gene An14g03280 encoding a putative UDP-glucose-4-epimerase seemed important as, it was recently published that the UDP-glucose-4-epimerase (UgeA) is needed for Gal*f* biosynthesis in *A. nidulans* [13]. The mutation in the *ugeA* (An14g03280) gene of mutant #41 (A to G) caused the change of a codon from AAC to GAC which consequently resulted in the change of Asn to Asp at position 191 in the UgeA protein. The mutation identified by the Illumina genome sequencing approach was confirmed by PCR amplification of the *ugeA* locus of the #41 strain followed by direct sequencing.

### Disruption of the *A. niger ugeA* gene and phenotypic analysis

To confirm that the mutation in An14g03820 (*ugeA*) caused the Gal*f*-low phenotype in mutant #41, a *pyrG* based gene deletion cassette was made. The deletion cassette (*pΔugeA::pyrG*) was transformed to MA169.4 and uridine prototrophic transformants were purified. During purification of the primary transformants it was found that most of the transformants displayed a reduced growth and a reduced sporulation phenotype, which resembled the phenotype of the #41 mutant. Subsequent diagnostic PCR and Southern blot analysis proved that the transformants with the growth phenotype contained an *ugeA* deletion (data not shown). Further phenotypic characterization of the *ΔugeA* mutant showed similar morphological alterations (increased branching and irregular length of hyphal compartments) as found for the #41 mutant (data not shown).

**Figure 4:**
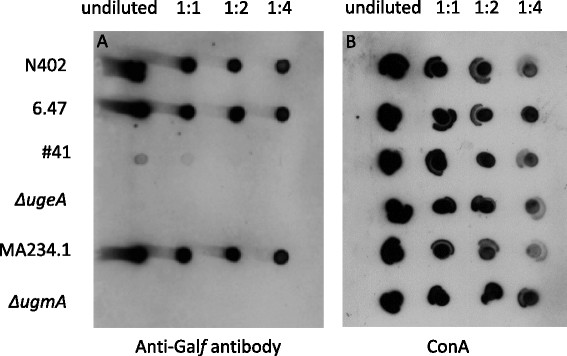
**Dot blot assay to detect the presence of Gal** ***f*** **residues on secreted glycoconjugates of different** ***A. niger*** **strains.** Strains were grown to early stationary phase and cell-free medium was spotted in a dilution series as indicated on nitrocellulose filter paper on two blots in parallel. The left blot was incubated with the anti-Gal*f* antibody (L10) the right blot was incubated with the ConA lectin.

**Figure 5:**
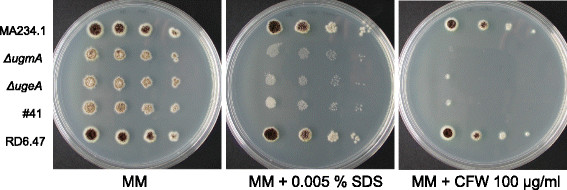
**Susceptibility of** ***A. niger*** **Gal** ***f*** **mutants towards SDS and CFW.** Ten-fold serial dilutions of spores derived from different Gal*f* mutants were spotted on minimal medium plates containing 0.005% SDS or 100 μg/ml CFW. Pictures were taken after three days of growth at 30°C.

As demonstrated above, very low levels of Gal*f* were observed in the dot blot (Figure 1) and Platelia assay (Figure 3A) for the #41 mutant. Interestingly, the amount of Gal*f* in the *ΔugeA* strain was strongly reduced compared to the #41 mutant, indicated by the complete lack of signal on the dot blot (Figure 4) and a further 10-fold reduction of the Gal*f*-signal in the Platelia assay (Figure 3A). These results suggest that the UgeA protein in #41 is still slightly active and able to convert some UDP-Glc*p* to UDP-Gal*p*, which is subsequently utilized for Gal*f*-biosynthesis. Despite the observation that more Gal*f* is present in the #41 mutant compared to the *ΔugeA mutant*, both mutants display similar phenotypes, although some subtle differences could be seen between the #41 and the *ΔugeA* mutants*.* The #41 mutant seems to sporulate slightly more intense on the MM plate and grows somewhat better on the 0.005% SDS plate (Figure 5).

**Figure 6:**
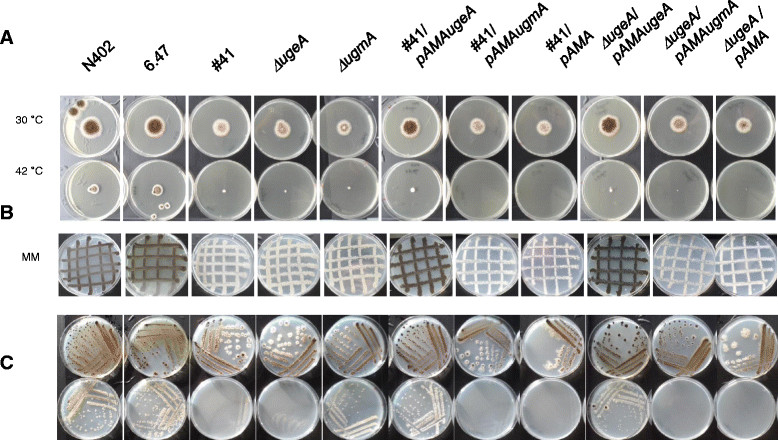
**Complementation analysis of the #41 mutant and the** ***ΔugeA*** **strain. A)** Complementation of the growth phenotype of the #41 and *ΔugeA* mutants by reintroducing the *ugeA* gene; **B)** Complementation of the sporulation defect in the #41 and *ΔugeA* mutants after transformation with the pAMAugeA vector; **C)** *ugeA* is required for growth on galactose and the inability to grow on galactose is restored by retransformation of the *ugeA* gene. Minimal medium plates in the upper row contain 25 mM glucose; lower row contain 25 mM galactose and 3 mM arabinose.

To further confirm that the mutation in ORF An14g03820 (*ugeA*) in the #41 mutant and the deletion of *ugeA* are the cause of the observed phenotypes, a complementation analysis was performed. Therefore, *pyrG*^−^ derivatives were obtained from the #41 and *ΔugeA* mutants by selecting on 5′FOA plates. The *ugeA* gene was PCR amplified and cloned into an autonomously replicating vector (pAMA-pyrG, [18]) and used for complementation. Transformation of the pAMA-*ugeA* plasmid to the #41 and *ΔugeA* mutants complemented the reduced growth and sporulation phenotype (Figure 6A and B) as well as the Gal*f*-negative phenotype in the dot blot analysis (data not shown). Control transformations with the empty plasmid (pAMA-*pyrG*) or the pAMA-*ugmA-pyrG* gene were also performed. In general, the transformation of these control plasmids did not complement the sporulation defect of the #41 and *ΔugeA* mutants. Unexpectedly, we occasionally (with a frequency of 0.2%-1%) obtained transformants with the empty or *ugmA* plasmid that sporulated well and grew like the complemented strain. From the original transformation plates of the *ΔugeA* mutant with the pAMA-pyrG or the pAMA-ugmA-pyrG plasmids, two transformants were purified with the improved sporulation phenotype. The pAMA-pyrG or pAMA-ugmA-pyrG transformant that sporulated well were also Gal*f-* positive in the dot blot analysis (data not shown). The reversion of the phenotype might be caused by second site suppressor mutations or by increased expression of redundant genes (see Discussion).

### UgeA is required for growth on galactose in *A. niger*

Based on sequence comparisons, UgeA (An14g03820) is predicted to encode a UDP glucose-4-epimerase and the result above clearly indicate that this gene is most likely responsible for the synthesis of UDP-Gal*p*, which is further converted to UDP-Gal*f* by the UgmA enzyme. *A. niger* contains in addition to UgeA (An14g03820) two other homologous genes predicted to encode UDP glucose-4-epimerases (An12g10410 and An02g11320). The presence of three putative UDP glucose-4-epimerases in *A. niger* is similar to *A. fumigatus* in which the function of the three genes (*uge3*, *uge5* and *uge4*) was recently analysed [12]. Based on homology, the *A. niger* UgeA is most similar to the *A. fumigatus* Uge5 protein and the UgeA protein of *A. nidulans* (See Table 1 for amino acid identities of the Uge homologs between the three species). To test whether the *A. niger ugeA* gene is required for growth on galactose, mutant #41, *ΔugeA* and their respective parental wild type strains were inoculated on plates containing 50 mM galactose and 0.3 mM arabinose. The supplementation of a second carbon source in addition to galactose is included because *A. niger* spores are unable to germinate on galactose alone [19]–[21]. As shown in Figure 6C, the #41 and *ΔugeA* mutants do not grow on galactose medium indicating that *ugeA* is required for growth on galactose. The inability to grow on galactose of the *ugeA* strain is not a consequence of its inability to produce Gal*f* as the *ugmA* deletion strain grows normally on galactose (Figure 6C).

**Table 1:** **Comparison of the percentage of amino acid identity between Uge proteins of** ***A. niger*** **,** ***A. fumigatus*** **, and** ***A. nidulans***

Species	Protein ID	***A. niger***			***A. fumigatus***			***A. nidulans***	
		UgeA An14g03820	UgeB An12g10410	UgeC An02g11320	Uge3 Afu3g07910	Uge4 Afu4g14090	Uge5 Afu5g10780	UgeA AN4727.2	UgeB AN2951.2
*A. niger*	UgeA (An14g03820)	100*	40	39	39	37	94	94	39
	UgeB (An12g10410)		100	66	58	59	41	39	58
	UgeC (An02g11320)			100	86	59	40	40	81
*A. fumigatus*	Uge3 (Afu3g07910)				100	58	39	39	84
	Uge4 (Afu4g14090)					100	37	39	56
	Uge5 (Afu5g10780)						100	94	37
*A. nidulans*	UgeA (AN4727.2)							100	39
	UgeB (AN2951.2)								100

* values indicate percentage of amino acids identities after pairwise alignment using BLASTP at http://blast.ncbi.nlm.nih.gov/Blast.cgi.

## Discussion

Screening of a collection of *A. niger* cell wall mutants, which were previously isolated by induced expression of the cell wall stress reporter gene (*agsA*), identified in total 9 mutants which did not secrete detectable levels of Gal*f*-containing glycoconjugates into the medium. Three mutants were identified previously [8] and six new Gal*f*-deficient mutants were additionally found in this screen. Interestingly, the mutants fell into two unevenly distributed complementation groups. A large group (represented by eight alleles) consisted of mutants in the *ugmA* gene and the other complementation group only contained a single representative with a mutation in the *ugeA* gene. The reason for this uneven distribution among the two complementation groups is not clear as both the *ugmA* and the *ugeA* mutants have very similar phenotypes when inactivated (Figures 4 and 5). Besides UgeA and UgmA, which are required for the synthesis of the UDP-Gal*f*, proteins involved in subsequent steps of the synthesis of Gal*f*-containing glycoconjugates have been recently identified and include a specific UDP-Gal*f* transporter (GlfB in *A. fumigatus* [5] and UgtA in *A. nidulans* [6] and a Gal*f*-transferase [7]. Mutants in the homologs of those genes in *A. niger* were not identified in our cell wall mutant collection. Analysis of the *A. niger* genome showed the presence of two homologs of GlfB/UgtA and three homologs of the GfsA protein (data not shown). The inability to detect representative mutants in the cell wall mutant collection suggests possible redundant functions of the pairs of genes in *A. niger*. Indeed, the two *A. niger* Gal*f* transporters only display a Gal*f* minus phenotype when both genes were deleted (Park and Ram, unpublished data).

This study represents the first example of the power of genome sequencing for characterizing classically obtained mutants in *A. niger*. The mutant #41 strain was created by UV mutagenesis which is known to preferably induce transitions (purine to purine (A-G) or pyrimidine to pyrimidine (T-C) mutations. From the 78 SNPs a majority of 49 (63%) SNPs were transitions and 29 (27%) were found to be transversions (purine to pyrimidine or reverse) (Additional file 1: Table S1). Of the 29 transversions, a high number of A to T transversions were found. The total number of mutations (21) in non-exonic regions was relative low taking into account the relative stringent dosis of UV treatment (66% survival). This number of SNPs makes it feasible to identify responsible mutants in the case that analysis of the predicted function of the mutated genes does not give a clear clue.

Interestingly, the Asn to Asp mutation at position 191 in UgeA demonstrated in mutant #41 seems to inhibit enzyme function greatly, but not completely. We aligned the amino acid sequences of UgeA from *A. niger* with that from *A. nidulans*, human and *Escherichia coli* (Additional file 3: Figure S1A). This shows that the Asn at position 191 is conserved between the Aspergilli and human, but not in *E. coli*. Recently, the complex crystal structure of UgeA from *A. nidulans* together with its substrates NAD + and UDP-Glc*p* was elucidated [22], which showed a similar orientation as in the human enzyme (GALE). This structure enabled us to visualize the location of Asn191 at the start of β–strand 6 (in red, Additional file 3: Figure S1B). Mutation of this residue to an Asp will result in the disruption of the ideal orientation of β–strands 6 and 7, and thereby of the substrate-orientation YFN-domain (residues 197–199). Apparently, this disruption is enough to diminish the UgeA enzyme activity greatly, resulting in lower amounts of available Gal*p* to flow into the Gal*f*-biosynthesis pathway.

This study shows that the *ugeA* gene of *A. niger* is required for UDP-Gal*f* biosynthesis and for growth on galactose. This is identical to the situation in *A. nidulans* in which it has been shown that the *ΔugeA* mutant also lacks Gal*f* and is unable to grow on galactose [13]. Sequence comparison showed that the *A. nidulans* UgeA protein is most similar to the UgeA protein of *A. niger* (Table 1). In *A. fumigatus*, the *uge5* mutant (Uge5 is the paralog of both *A. niger* and *A. nidulans* UgeA protein) can grow on galactose medium. It has recently been shown that the *A. fumigatus* Uge3 protein is responsible for the growth on galactose as disruption of both *uge3* and *uge5* results in a galactose auxotrofic strain [12]. The galactose auxotrophy of the *A. niger ΔugeA* and *A. nidulans ΔugeA* mutants either indicated that the Uge3 homologs which are present in the genome are not expressed or have a more specific activity. The Uge3 protein of *A. fumigatus* has been shown to be involved in galactosaminogalactan synthesis and can mediate both the production of UDP- galactose and UDP-*N*-acetyl-galactosamine. Further enzymatic and molecular genetic analysis of the Uge homologs in *A. niger* and *A. nidulans* is required to understand their exact function and their possible role in galactosaminogalactan biosynthesis. In this respect, it is interesting that we observed high frequency of suppression of the *ΔugeA* phenotype after transformation. Although the phenotype of the #41 and *ΔugeA* mutants were stable during normal propagation, transformation of both strains yielded regularly suppressor mutations that were restored in their ability to produce Gal*f*-decorated galactomannoproteins and were also restored in their ability to grow on galactose (data not shown). A possible mechanism to explain the development of suppressors include activation of expression of one of the *uge* genes, or occurrence of a mutation in one of these genes that changes substrate specificity.

## Conclusion

By screening a collection of UV-generated cell wall mutants, a new Gal*f*-deficient mutant was identified. We used whole genome sequencing to identify the mutation responsible for this mutant phenotype. Complementation and targeted deletion studies confirmed that the UDP-glucose-4-epimerase gene (*ugeA*) is required for the biosynthesis of Gal*f* as well as for galactose metabolism in *Aspergillus niger*.

## Methods

### Strains and culture conditions

The *Aspergillus niger* strains used in this study are listed in Table 2. Strains were grown on minimal medium (MM) [23] containing 1% (wv-1) glucose as carbon source or complete medium (CM) containing 0.5% (wv-1) yeast extract, 0.1% (wv-1) casamino acids in addition to MM. When required, plates were supplemented with 10 mM uridine. 5′FOA selection for the selection of *pyrG*^−^ strains was performed as described previously [24]. For the plate growth assays, strains were grown on MM plates containing 25 mM glucose, 25 mM galactose, or 25 mM galactose and 3 mM arabinose. For the heterokaryon assay, MM-plates were supplemented with 0.005% SDS.

**Table 2:** ***Aspergillus niger*** **strains used in this study**

Strain	Description	References
N402	*cspA1* derivative of ATCC9029	Bos *et al.* 1988
MA70.15	Δ*kusA::amdS, pyrG*-	[25]
MA169.4	Δ*kusA::DR_amdS_DR, pyrG*-	[18]
MA234.1	MA169.4 transformed with pAB4.1 to make strain *pyrG+*	Arentshorst, unpublished
RD6.47	pPagsA-amdS-TamdS-pyrG* and p*PagsA-H2B-GFP-TtrpC*/pAN7.1	[8]
RD6.47#41	*galF*- mutant derived from RD6.47	[8]
RD6.47#41, *pyrG*-	*pryG-* derivative of RD6.47#41	This study
RD6.47#41/pAMA_*ugeA*	RD6.47#41/*pyrG*- p*Blue_AMA_ugeA*	This study
RD6.47#41/pAMA_*ugmA*	RD6.47#41/*pyrG*- p*Blue_AMA_ugmA*	This study
RD6.47#41/pAMA	RD6.47#41/*pyrG*- p*Blue_AMA*	This study
JH12.1	*ugeA* (An14g03820)::*pyrG* deletion in MA169.4	This study
JH21.1	*pyrG-* derivative of JH12.1	This study
JH21.1.1	Δ*ugeA*/pAMA_*ugeA*	This study
JH21.1.2	Δ*ugeA*/pAMA_*ugmA*	This study
JH21.1.3	Δ*ugeA*/pAMA	This study
MA87.6	*ugmA* (An02g08660)::*pyrG* in MA70.15	[8]
MA247.2.1b	*pryG-* derivative of MA87.6	This study
MA247.2.1b.1	Δ*ugmA*/pAMA_*ugeA*	This study
MA247.2.1b.2	Δ*ugmA*/pAMA_*ugmA*	This study
MA247.2.1b.3	Δ*ugmA*/pAMA	This study
RD15.4	pPagsA-H2B-GFP-TtrpC-pyrG* and p*PagsA-amdS-TamdS*/pAN7.1	[8]
RD15.4#5	*galF*- mutant derived from RD15.4	[8]
RD15.4#18	*galF*- mutant derived from RD15.4	[8]
RD15.4#30	*galF*- mutant derived from RD15.4	[8]
RD15.4#50	*galF*- mutant derived from RD15.4	[8]
RD15.4#57	*galF*- mutant derived from RD15.4	[8]
RD6.13#44 (*miaA*)	*ugmA* mutant derived from RD6.13	[8]
RD15.4#17 (*miaB*)	*ugmA* mutant derived from RD15.4	[8]
RD6.13#50 (*miaC*)	*ugmA* mutant derived from RD6.13	[8]

*pyrG+* = *pyrG* plus (uridine prototroph); *pyrG-* = *pyrG* minus (uridine auxotroph); pyrG* = mutant *pyrG* allele for targeting at the *pyrG* locus).

### Screening methods to identify Gal*f* minus mutants

Strains from the collection of 240 cell wall mutants of *A. niger* [8] were grown in 25 ml CM in 50 ml tube Greiner tube for 24 h at 30°C. Medium samples were filtered over a Whatman glass microfiber filter and 2 μl medium was spotted on nitrocellulose blotting paper. Blots were incubated with the L10 monoclonal anti-Gal*f*-antibody (1:10) in TSMT (TSM [20 mM Tris–HCl, pH 7.4, 150 mM NaCl, 2 mM CaCl_2_, 2 mM MgCl_2_] with 0.05% Tween-20) supplemented with 5% BSA or with peroxidase labeled ConA (ConA-PO EY Laboratories, USA) used in a 1:500 dilution in PBS containing 0.05% (v/v) Tween-20. Blots with the L10 antibody were subsequently incubated with rabbit-a-mouse-HRP (PO161, DAKO) as a secondary antibody in TSMT with 5% BSA. For detection of the secondary antibody or the ConA-PO conjugate, a chemiluminescence kit (Thermo Scientific Pierce) was used.

### Isolation of cell wall galactomannan

Cell walls were isolated from *A. niger* wild type strain N402 and the #41 mutant strain after growth in CM for 24 h at 30°C at 250 rpm. Spores (1 × 10^9^) were inoculated in 1 L of CM in a 2 L Erlenmeyer. The mycelium of both strains was isolated by filtering over myracloth and grinded in liquid nitrogen using a pestle and mortar and the broken mycelia washed 3 times with 1 M NaCl and 3 times with MilliQ at 4°C by centrifugation (3600 rpm, 10 min). Successful breakage of the mycelia was confirmed by microscopy. Isolated cell walls were lyophilized for 24 h using 1.0 gram of cell walls (dry weight) for both the N402 and the #41 strain. The galactomannan fraction was isolated according to Bardalaye and Nordin [16]. The yield of purified galactomannan was 21.2 mg and 10.4 mg from 1 gram of freeze dried cell walls for the N402 and #41 strain, respectively.

### Platelia assay

Microtiter plate wells containing coated antibody EB-A2, which recognizes Gal*f*-moieties on galactomannan [14] were filled with 50 μl EB-A2 conjugated to HRP (all from Platelia Aspergillus EIA kit, Bio-Rad). Supernatant of *A. niger* strains or purified galactomannan was added to the wells and incubated for 90 min at 37°C. After washing 5 times, 200 μl 3,3′,5,5′-tetramethylbenzidine (TMB) detection mixture was added and samples were incubated for 30 min at ambient temperature in the dark. After stopping the coloring reaction by addition of 100 μl 1.5 N H_2_SO_4_, relative amounts of Gal*f* on galactomannan were determined by measuring the optical density (OD) by spectrophotometry at 450 nm.

### Monosaccharide analysis by HPAEC

Galactomannan isolated from *A. niger* was analyzed for its monosaccharide composition by High-Performance Anion Exchange Chromatography (HPAEC), using the method described by [26] with minor modifications. In summary, chromatography of the samples was performed in a Dionex, Bio-LC system, using a CarboPac PA 10 column (250 ×2 mm) in combination with a CarboPac guard column (2×50mm), Dionex Corp. Around 2 mg galactomannan was dissolved in 2 ml water and incubated with 360 μl of TFA for 5 h at 100°C. Hereafter, 3 ml MilliQ water was added and samples were freeze dried overnight. The pellets were dissolved in 100 μl water. After diluting 1:100, 20 μl (equivalent to 4 μg polysaccharide) was injected onto the column. Monosaccharides were eluted isocratically using H_2_O and 1 M NaOH in a ratio of 80:20 for 30 min. The column was washed with water for 10 min and equilibrated with 250 mM NaOH for 10 min before every injection.

### Western blot analysis

Medium samples of the various strains were obtained as described for the dot-blot analysis and 20 ul of culture filtrate was used for Western blot analysis. SDS-PAGE and blotting were carried out as described [27]. For labeling with the anti-Gal*f* antibody (L10), the membrane was blocked with 5% low-fat milk in TTBS (Tris-buffered saline, 0.05% Tween-20), and Gal*f* was detected using the L10 antibody (1/5,000) overnight, followed by a goat anti-rabbit horseradish peroxidase secondary antibody (1/20,000) for 1 h. Detection was performed using a chemiluminescence kit (Thermo Scientific Pierce), according to manufacturer’s instructions.

### Genetic methods

Fungal transformations were done according the protoplast method described by [24]. Complementation tests were done using the heterokaryon test. 2 ul of spores (1 × 10^6^ spores/ml) of strains to be tested were inoculated on MM plates containing 0.005% SDS approximately 1 cm apart from each other. Plates were incubated for 3 days at 30°C and analyzed for a zone of conidiation of heterokaryotic mycelium.

### Plasmid construction

For the construction of the *ugeA* deletion cassette the Multisite GatewayR Three-Fragment Vector Construction kit was used. As a marker for deleting the *ugeA* gene, the *pyrG* marker of *A. oryzae* was used. To facilitate removal of the AopyrG marker, *A. nidulans* tTrpC repeats were cloned around the *pyrG* gene [28]. The TrpC-pyrG-TrpC fragment was PCR amplified using primer listed in Table 3 and cloned in pDonor221. The 5′ and 3′ flanking regions of *ugeA* were PCR amplified using primers with appropriate attB sites (Table 3) and the 799 bp fragments were cloned into pDONR P4-P1R and pDONR P2R-P3 respectively. The subsequent LR reaction was performed using pDONR_ugeA5, pDONR_ugeA3 and pDONR_TrpC_pyrG_TrpC and pDEST R4-R3 Vector 2 to create the *ugeA* deletion plasmid. The final construct was verified by restriction analysis and sequencing.

**Table 3:** **The primers used in this study**

Name	Sequence 5′-3′	Aplication
attB1_TrpC_CFP_F	*ggggacaagtttgtacaaaaaagcaggct* ATGGACGAGCTGTACAAGTAA	Amplification TrpC-pyrG-TrpC cassette
attB2r_TrpC_R	*ggggaccactttgtacaagaaagctgggt* TGGGTGTTACGGAGCATTCACTAGGC	Amplification TrpC-pyrG-TrpC cassette
attB4_ugeA5F	*ggggacaactttgtatagaaaagttg* CCGATAGGAAGGATGAGGAT	Amplification 5′flank *ugeA* disruption cassette
attB1r_ugeA5R	*ggggactgcttttttgtacaaacttg* GATGATGATAAGGTATGACT	Amplification 5′flank *ugeA* disruption cassette
attB2r_ugeA3F	*ggggacagctttcttgtacaaagtgg* ATAATGACCCCGCATATGTT	Amplification 3′flank *ugeA* disruption cassette
attB3_ugeA3R	*ggggacaactttgtataataaagttg* GGATGGACAGCCGTGCAGTG	Amplification 3′flank *ugeA* disruption cassette
ugmAP1f-NotI	aaggaaaaaa**gcggccgc** AGGACTCCATAGGCCCGTAGA	Amplification *ugmA* for complementation and sequencing
ugmAP2r-NotI	aaggaaaaaa**gcggccgc** AGAAACGGACTGCATGGGC	Amplification *ugmA* for complementation and sequencing
ugeAfw-SmaI	ctcgag**cccggg** TTGATTGGACCCTTGGGATCG	Amplification *ugeA* for complementation and sequencing
ugeArev-SmaI	ctcgag**cccggg** TTGGCAAGGAAGGAGGTGAAG	Amplification *ugeA* for complementation and sequencing

Italic letters indicate attB sites, restriction sites are shown in bold.

For the complementation analysis the autonomously replicating vector pMA172 was used [18]. The *ugeA* or *ugmA* genes including promoter and terminator regions were PCR amplified using primers listed in Table 3, cloned into pJet2.1 and verified by sequencing. The *ugeA* and *ugmA* genes were reisolated as *Sma* I (*ugeA*) or *Not* I (*ugmA*) fragments and cloned into the unique *Sma* I or *Not* I site of pMA172 to give pAMA-ugeA and pAMA-ugmA respectively.

### Genome sequencing and SNP identification

Genomic DNA isolations of strains 6.47 (parental strain) and #41 (mutant strain) were performed as described [24]. Genomic DNA was further purified using Macherey-Nagel NucleoBond Xtra columns. The Illumina Paired-End sequencing was performed by ServiceXS using Illumina kits (cat# 1001809 and 1005063) and protocols according to the instructions by the supplier. The quality and yield after sample preparation were checked and were consistent with the expected size of 300 bp after excision from gel. Clustering and DNA sequencing using Illumina cBot and HiSeq 2000 was performed according to manufacturer’s protocols. A total of 6.5 pmol of DNA was used. Two sequencing reads of 100 cycles each using Read1 sequencing and Read2 sequencing primers were performed with the flow cell. For strains 6.47 and #41, 3.1 and 3.3 Gb of DNA sequence was obtained respectively. The genome sequences are available on request. Image analysis, base calling and quality check was performed with the Illumina data analysis pipeline RTA v1.13.48 and/or OLB v1.9/CASAVA v1.8.2. SNPs between the two strains (mutant #41 and parental strain 6.47), were identified using *A. niger* strain ATCC1015 (http://genome.jgi-psf.org/pages/search-for-genes.jsf?organism=Aspni5) as a reference genome. Low quality bases were removed from the raw sequencing data. A Q25 phred score was used as a minimum and bases with phred scores below were removed and reads containing these bases were split. If the resulting reads from splits were shorter than 40 bases they were removed altogether. Alignment of filtered reads was performed with BWA (version 0.5.9) which lays the foundation for finding SNPs. For each SNP it was verified whether the SNP was in a predicted protein encoding region using the *A. niger* 3.0 genome at JGI using the SNP coordinates.

## Availability of supporting data

The data set(s) supporting the results of this article are included within the article and its additional files. Genome sequences of RD6.47 and mutant #41 are available upon request.

## Additional files

## Electronic supplementary material

Additional file 1: Tabel S1.: List of all SNPs identified in mutant #41. (XLS 49 KB)

Additional file 2: Tabel S2.: SNPs in coding regions in #41. (DOCX 25 KB)

Additional file 3: Figure S1.: The Asn to Asp mutation at position 191 in mutant #41 probably leads to misorientation of the substrate-orientation YFN-domain in UgeA. A) Protein alignment of the UgeA homologues from *A. niger*, *A. nidulans*, human and *E. coli*. Residues interacting with carbohydrate substrate are indicated with a black star, residues interacting with NAD substrate are indicated with a yellow star, the mutation identified in mutant #41 (N191D) is indicated with the blue arrow. B) Cartoon representation of the crystal structure from *A. nidulans* (PDB ID: 4LIS, [22], with β − strands 6 and 7 in red, residues interacting with carbohydrate substrate in black C) Space-filling model of UgeA; showing that the key enzymatic residues are located on the inside of UgeA D) Space-filling model of selected amino acid residues of UgeA: N191 in blue, residues forming β − strands 6 and 7 in red, residues interacting with carbohydrate substrate in black. (PPTX 891 KB)

Below are the links to the authors’ original submitted files for images.

Authors’ original file for figure 1

Authors’ original file for figure 2

Authors’ original file for figure 3

Authors’ original file for figure 4

Authors’ original file for figure 5

Authors’ original file for figure 6
