# Supplementary material for: Identification of the UDP-glucose-4-epimerase required for galactofuranose biosynthesis and galactose metabolism in A. niger
Source: Fungal Biol Biotechnol. 2014 Oct 14;1:6. doi: 10.1186/s40694-014-0006-7 (PMC5598270; doi:10.1186/s40694-014-0006-7)
Supplement: Supplementary file 2 — Additional file 2: Tabel S2.: SNPs in coding regions in #41. (DOCX 25 KB) [file 40694_2014_6_MOESM2_ESM.docx]

Suppl. Tabel 2. SNPs in coding regions in #41

| Chromosome | Location | mutantion and orientation OFR | ORF | SNP | Gene ID | Description ASPGD | Mutation | Amino acid changed |
| --- | --- | --- | --- | --- | --- | --- | --- | --- |
| chr_1_1 | 373467 | C->G (-) | 1 | Mut#5 | An09g01560 | Has domain(s) with predicted transferase activity, transferring glycosyl groups activity and membrane localization | atG-atC | Met-Ile |
| chr_1_2 | 456268 | C->T (+) | 2 | Mut#10 | An14g01760 | strong similarity to 3-oxoacyl-[acyl-carrier protein] synthase | Ccg-Tcg | Pro-Ser |
| chr_1_2 | 980036 | T->C (-) | 3 | Mut#11 | An14g03820 | strong similarity to UDP-glucose 4-epimerase; Leloir pathway enzyme | Aac-Gac | Asn-Asp |
| chr_1_2 | 1422105 | T->A (-) | 4 | Mut#12 | An14g05970 | Ortholog(s) have Golgi apparatus, fungal-type vacuole membrane localization, unknown function | Aac-Tac | Asn-Tyr |
| chr_2_1 | 351540 | G->A (+) | 5 | Mut#13 | An13g01320 | Protein with unknown function. | Gat-Aat | Asp-Asn |
| chr_2_2 | 966040 | T->A (+) | 6 | Mut#18 | An01g03450 | Protein with unknown | taT-taA | Tyr-*STOP* |
| chr_3_1 | 10395 | A->T (-) | 7 | Mut#24 | An02g11480 | Strong similarity with nitrogen regulatory protein tamA | GTc-AAc | Val-Asn |
| chr_3_1 | 10396 | C->T (-) |  | Mut#25 |  |  |  |  |
| chr_3_2 | 319845 | G->A (-) | 8 | Mut#29 | An12g03660 | Strong similarity to protein C-terminal S-isoprenylcysteine carboxyl O-methyltransferase activity | cCC-cTT | Pro-Leu |
| chr_3_2 | 319846 | G->A (-) |  | Mut#30 |  |  |  |  |
| chr_3_2 | 836674 | C->T (-) | 9 | Mut#31 | An12g05740 | Protein with unknown, has domain(s) with predicted nucleoside-triphosphatase activity | Gat-Aat | Asn-Asp |
| chr_3_4 | 363088 | A->G (-) | 10 | Mut#32 | An15g01230 | Protein with unknown function, similarity to GTPase activating protein(Tsc2) | tTa-tCa | Leu-Ser |
| chr_3_4 | 935034 | A->G (-) | 11 | Mut#33 | An15g03820 | Protein with unknown function has domain(s) with predicted choline dehydrogenase activity | Tcg-Ccg | Ser-Pro |
| chr_4_1 | 1503576 | A->G (-) | 12 | Mut#36 | An04g09660 | Protein with unknown function, has domain(s) with predicted oxidoreductase activity | GTg-TCg | Val-Ser |
| chr_4_1 | 1503577 | C->A (-) |  | Mut#37 |  |  |  |  |
| chr_4_1 | 1607404 | A->G (+) | 13 | Mut#39 | An07g07210 | Ortholog(s) have protein serine/threonine kinase activity | Gat-Aat | Asn-Asp |
| #41 | chr_4_2 | A->G (+) | 14 | Mut#41 | An16g06440 | C2H2 Zn-finger transcription factor with unknown function | cAa-cGa | Gln-Arg |
| chr_5_3 | 390900 | T->C (-) | 15 | Mut#50 | An16g08180 | Ortholog(s) have protein serine/threonine kinase  activity | caA-caT | Gln-His |
| chr_7_1 | 1194930 | C->T (+) | 16 | Mut#57 | An11g04480 | Protein with similarity to non-specific RNA polymerase II transcription factor Srb9 | cCC-cTT | Pro-Leu |
| chr_7_1 | 1194931 | C->T (+) |  | Mut#58 |  |  |  |  |
| chr_8_1 | 1443479 | C->T (+) | 17 | Mut#63 | An18g06110 | strong similarity to regulator of G protein signaling domain protein (RgsA) | aCt-aTt | Thr-Ile |
| chr_8_1 | 1526234 | A>T (-) | 18 | Mut#64 | An18g06520 | strong similarity to protein involved in cell wall biogenesis and architectur e Ecm29 | ctA-cGa | Leu-Arg |
| chr_8_2 | 72664 | C->T (+) | 19 | Mut#65 | An08g00420 | Strong similarity to cytosolic iron-sulfur protein assembly protein 1 | tCg-tTg | Ser-Leu |
| chr_8_2 | 1915530 | C->T (+) | 20 | Mut#69 | An08g08020 | similarity to bialaphos acetylhydrolase bah - Streptomyces hygroscopicus | Caa-Taa | Gln-*stop* |
| chr_8_2 | 2809445 | C->T (-) | 21 | Mut#74 | An06g01130 | strong similarity to kinesin heavy chain Nkin - Neurospora crassa | Gag-Aag | Glu-Lys |
