# Supplementary figures and images for: Identification of the UDP-glucose-4-epimerase required for galactofuranose biosynthesis and galactose metabolism in A. niger
Source: Fungal Biol Biotechnol. 2014 Oct 14;1:6. doi: 10.1186/s40694-014-0006-7 (PMC5598270; doi:10.1186/s40694-014-0006-7)

## Slide 1
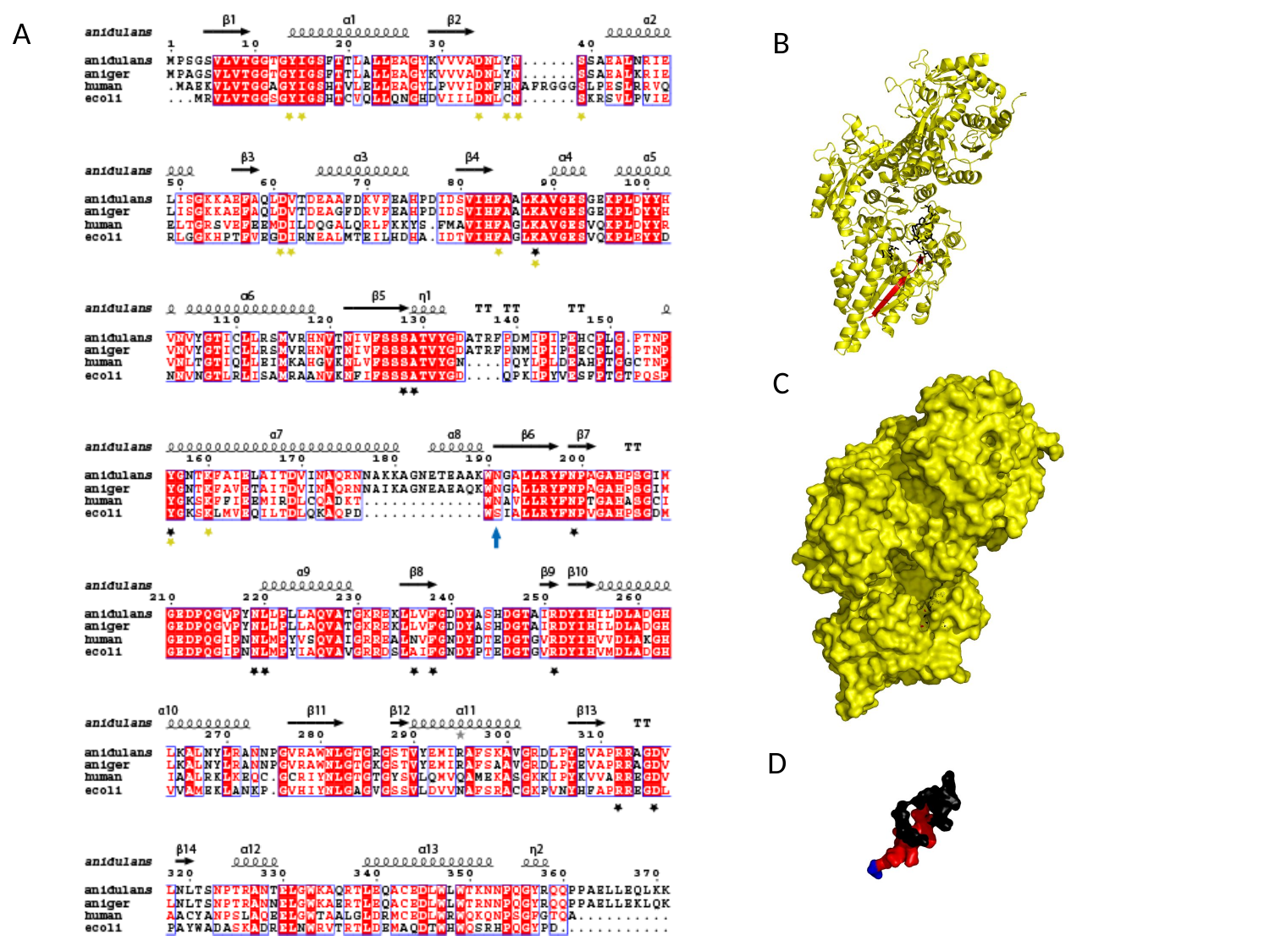

A
B
C
D

Supplement: Supplementary file 3 — Additional file 3: Figure S1.: The Asn to Asp mutation at position 191 in mutant #41 probably leads to misorientation of the substrate-orientation YFN-domain in UgeA. A) Protein alignment of the UgeA homologues from A. niger, A. nidulans, human and E. coli. Residues interacting with carbohydrate substrate are indicated with a black star, residues interacting with NAD substrate are indicated with a yellow star, the mutation identified in mutant #41 (N191D) is indicated with the blue arrow. B) Cartoon representation of the crystal structure from A. nidulans (PDB ID: 4LIS, [22], with β − strands 6 and 7 in red, residues interacting with carbohydrate substrate in black C) Space-filling model of UgeA; showing that the key enzymatic residues are located on the inside of UgeA D) Space-filling model of selected amino acid residues of UgeA: N191 in blue, residues forming β − strands 6 and 7 in red, residues interacting with carbohydrate substrate in black. (PPTX 891 KB) [file 40694_2014_6_MOESM3_ESM.pptx]

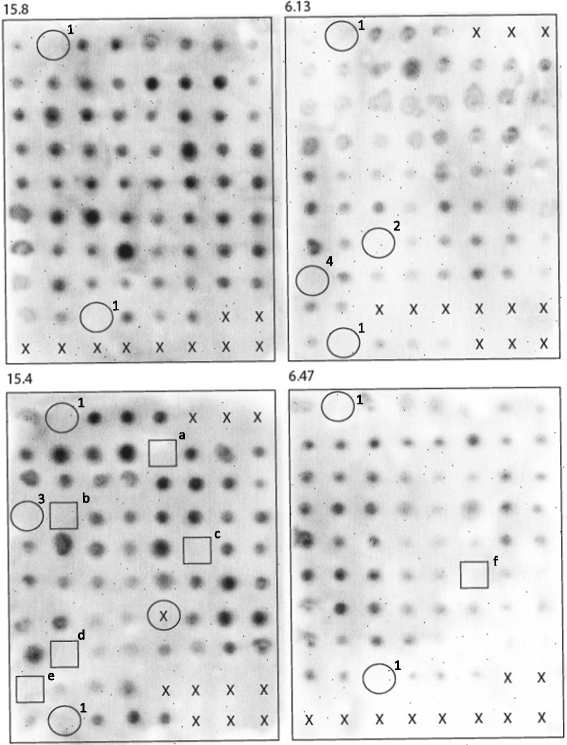

Supplement: Supplementary file 4 — Authors’ original file for figure 1 [file 40694_2014_6_MOESM4_ESM.gif]

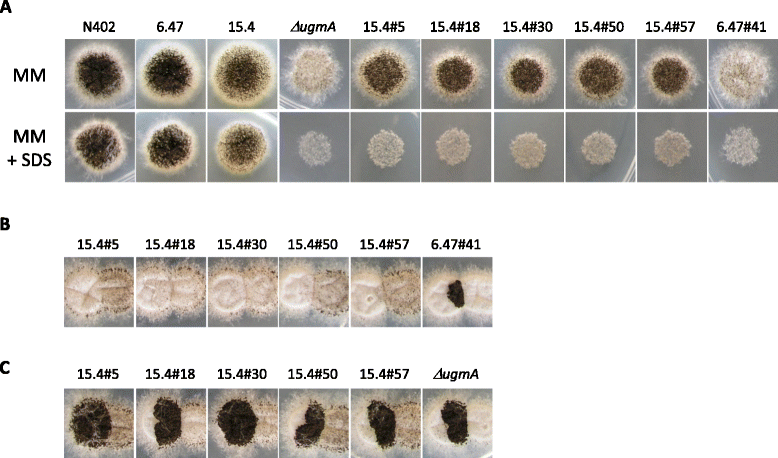

Supplement: Supplementary file 5 — Authors’ original file for figure 2 [file 40694_2014_6_MOESM5_ESM.gif]

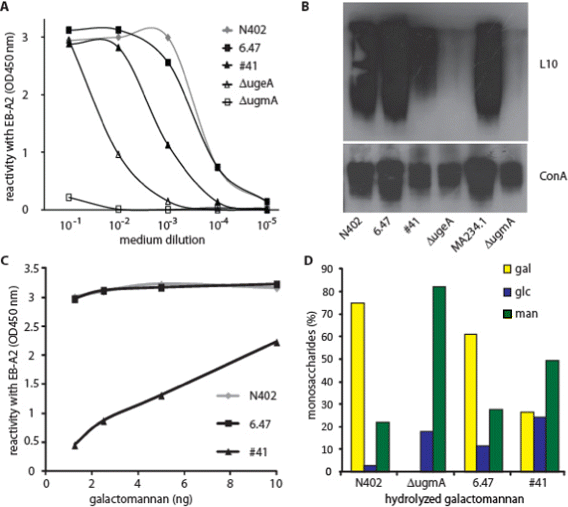

Supplement: Supplementary file 6 — Authors’ original file for figure 3 [file 40694_2014_6_MOESM6_ESM.gif]

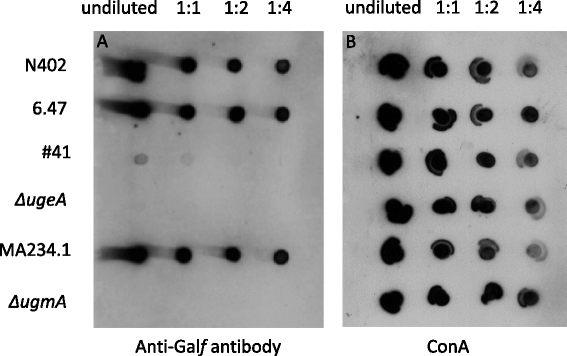

Supplement: Supplementary file 7 — Authors’ original file for figure 4 [file 40694_2014_6_MOESM7_ESM.gif]

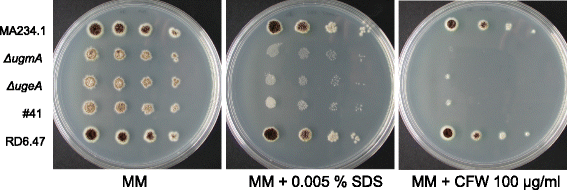

Supplement: Supplementary file 8 — Authors’ original file for figure 5 [file 40694_2014_6_MOESM8_ESM.gif]

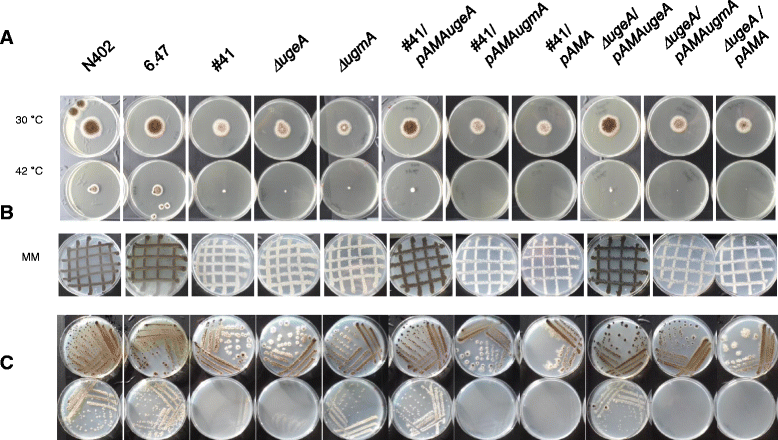

Supplement: Supplementary file 9 — Authors’ original file for figure 6 [file 40694_2014_6_MOESM9_ESM.gif]
